# Antimicrobial peptides extend lifespan in *Drosophila*

**DOI:** 10.1371/journal.pone.0176689

**Published:** 2017-05-17

**Authors:** Gerrit Loch, Ingo Zinke, Tetsushi Mori, Pilar Carrera, Jonas Schroer, Haruko Takeyama, Michael Hoch

**Affiliations:** 1 Molecular Developmental Biology, Life & Medical Sciences Institute (LIMES), University of Bonn, Bonn, Germany; 2 Molecular Brain Physiology and Behavior, Life & Medical Sciences Institute (LIMES), University of Bonn, Bonn, Germany; 3 Faculty of Science and Engineering, Waseda University, Tokyo, Japan; University of Mississippi, UNITED STATES

## Abstract

Antimicrobial peptides (AMPs) are important defense molecules of the innate immune system. High levels of AMPs are induced in response to infections to fight pathogens, whereas moderate levels induced by metabolic stress are thought to shape commensal microbial communities at barrier tissues. We expressed single AMPs in adult flies either ubiquitously or in the gut by using the inducible GeneSwitch system to tightly regulate AMP expression. We found that activation of single AMPs, including *Drosocin*, resulted in a significant extension of *Drosophila* lifespan. These animals showed reduced activity of immune pathways over lifetime, less intestinal regenerative processes, reduced stress response and a delayed loss of gut barrier integrity. Furthermore, intestinal *Drosocin* induction protected the animals against infections with the natural *Drosophila* pathogen *Pseudomonas entomophila*, whereas a germ-reduced environment prevented the lifespan extending effect of *Drosocin*. Our study provides new insights into the crosstalk of innate immunity, intestinal homeostasis and ageing.

## Introduction

Animals and plants use antimicrobial peptides (AMPs) as a first line of defense to limit infections [1]. AMPs are small (six to 100 amino acids), cationic and amphipathic peptides of variable sequence and structure. They exhibit broad-spectrum activity against all kinds of microorganisms including bacteria, fungi, parasites and also viruses [2].

In mammals AMPs are expressed in many cell types including immune cells and cells of epithelial barriers like skin and gut [3]. As just one example, in humans down regulation of alpha-defensins is associated with Crohn’s disease, a chronic inflammatory bowel disease [4]. In addition to being involved in the pathogen defense, recent studies have shown that AMPs also play a pivotal role in intestinal homeostasis by regulating the composition and abundance of the gut microbiota [4]. Analyzing functions of AMPs is thus of major interest to biomedical research and to this end we have used *Drosophila* as a genetic model system.

In *Drosophila* seven distinct families of inducible AMPs have been identified to date. In response to an infection, they can be expressed systemically in the fat body or locally in epithelial barrier tissues [5]. AMP expression is regulated by members of the nuclear factor-kappa B family of inducible transactivators, which include the dorsal-related immunity factor (DIF), Relish and Dorsal. These transcription factors are activated upon infection by two major signaling cascades, the Toll and immune deficiency (IMD) pathways [5]. Additionally, subsets of AMPs can be directly activated by the transcription factors *Drosophila* Forkhead box O (dFOXO) or Forkhead (FKH), depending on the metabolic status of the fly, demonstrating a cross regulation between metabolism and innate immunity [6,7]. In the midgut AMP expression is not regulated by Toll signaling but by the IMD and the Janus kinase-signal transducers and activators of transcription (JAK-STAT) pathways [8] and controlled by the negative transcriptional regulator caudal [9]. It has been described that this local AMP expression at the intestinal barrier is necessary to fight food-borne infections, demonstrated with the *Drosophila* pathogen *Pseudomonas entomophila* (*Pe*) [10,11]. Additionally, the *Drosophila* intestinal epithelium is not only challenged by pathogenic bacteria, but is also as a host in constant contact to a commensal microbiota [12], which has to be regulated in composition and density by the immune system [13]. Both, pathogenic bacteria and the commensal microbiota have a tremendous influence on the intestinal homeostasis, a condition regulated by the immune and stress responses as well as the regenerative activity of the epithelial tissue [8,9,14]. The integrity of the intestinal barrier epithelium and the homeostasis of the gut are therefore also tightly linked to organismal health and lifespan [15,16]. In this study we used the fruit fly *Drosophila melanogaster* to analyze the interplay between intestinal AMP activation, midgut homeostasis and longevity.

## Results

### *Drosocin* and *CecropinA1* expression extends lifespan

**Fig 1:**
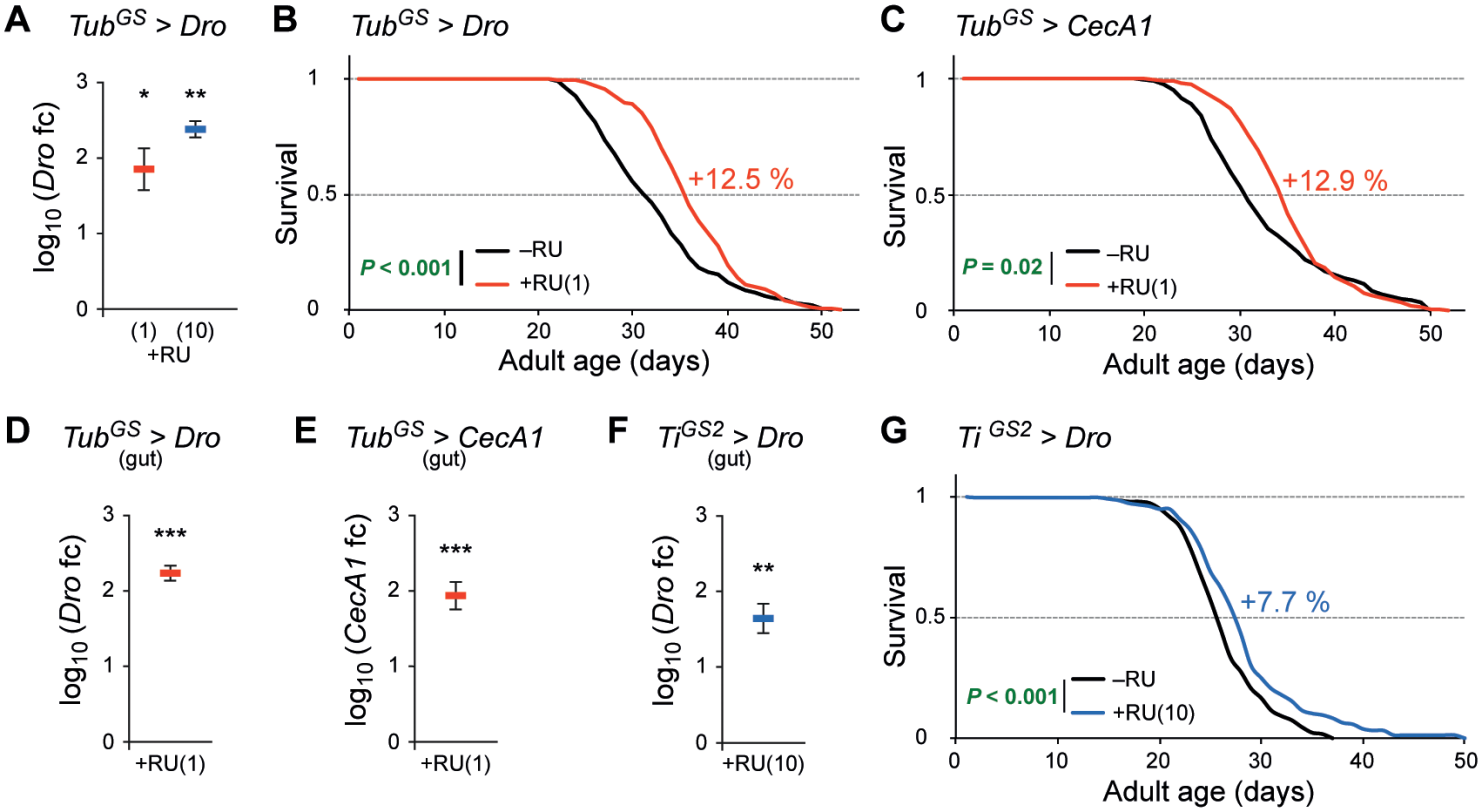
AMP expression extends lifespan. (A) *Dro* expression with the *tubulin*-GeneSwitch driver is dependent on the RU486 concentration. qPCR analysis of *Dro* in 12-day old female *Tub*^*GS*^*>Dro* flies. Expression was induced with 1 μg/ml (red) or 10 μg/ml (blue) RU. *n* = 3 replicates of four whole flies. (B–C) Ubiquitous expression of AMPs extends lifespan. Compared to controls fed without RU (–RU, black line), female *Tub*^*GS*^*>Dro* (B) or *Tub*^*GS*^*>CecA1* (C) flies fed with 1 μg/ml RU (+RU, red line) show 12.5% or 12.9% increased MLS, respectively. *n* ≥ 220 (B), *n* ≥ 191 (C) flies/condition. (D–E) *Dro* (D) and *CecA1* (E) transcription in the midgut of 14-day old female *Tub*^*GS*^*>Dro* (D) and *Tub*^*GS*^*>CecA1* (E) flies fed with 1 μg/ml RU (red). *n* = 4 (D), *n* = 5 (E) replicates of five midguts. (F–G) Gut specific expression of *Dro* extends lifespan. (F) *Dro* transcription in the midgut of 14-day old female *Ti*^*GS2*^*>Dro* flies fed with 10 μg/ml RU (blue). *n* = 4 replicates of five midguts. (G) Compared to controls (–RU, black line) female *Ti*^*GS2*^*>Dro* flies fed with 10 μg/ml RU (+RU, blue line) show 7.7% increased MLS. *n* ≥ 169 flies/condition. In (A) and (D–F) mean *log*_*10*_ of fold change is compared to controls (set to 0, not shown). Statistical tests: (A), (D–F) one-sample t-test, (B–C), (G) log-rank test (Kaplan-Meier analysis). * *P* ≤ 0.05, ** *P* ≤ 0.01, *** *P* ≤ 0.001. Error bars represent the standard error of the mean. *Dro*, *Drosocin*; *CecA1*, *Cecropin A1*; MLS, median lifespan; +RU, RU treatment. Genotypes were: *w/y*,*w;UAS-Dro/+;tubulin*^*GeneSwitch*^*-gal4/+* (*Tub*^*GS*^*>Dro*), *w/y*,*w;+/+;tubulin*^*GeneSwitch*^*-gal4/UAS-CecA1* (*Tub*^*GS*^*>CecA1*), *w/y*,*w;UAS-Dro/+;TiGS2*^*GeneSwitch*^*-gal4/+ (Ti*^*GS2*^*>Dro)*.

We used the RU486-inducible GeneSwitch system for spatial, temporal and gradual transcription [17,18] of several single AMPs in mated female flies. Ubiquitous activation of an *UAS-Drosocin* construct with a GeneSwitch GAL4 driver under the control of the *α-tubulin84B* promoter (*Tub*^*GS*^) [19] showed a successful and RU486 (RU) concentration dependent induction of *Drosocin* (*Dro*) transcription levels, analyzed by quantitative *real time* PCR (qPCR) (Fig 1A). As an appropriate level of AMP gene activity might be critical for a positive lifespan effect, we tested RU concentrations of 0.5, 1 and 10 μg/ml in longevity experiments. Inducing *Dro* with 1 μg/ml RU significantly extended the lifespan in two independent experiments and increased the median lifespan (MLS) by 12.5% (Fig 1B) and 10.0% respectively (S1A Fig). A positive result was also obtained by inducing *Dro* with an RU concentration of 0.5 μg/ml, leading to an increased MLS of 6.7% (S1B Fig). Using a higher concentration 10 μg/ml RU did not lead to a significant effect on lifespan (S1C Fig).

A significant positive effect on *Drosophila* lifespan was also achieved by inducing *CecropinA1* (*CecA1*) expression with 1 μg/ml RU in *Tub*^*GS*^*>CecA1* flies. In two independent experiments the MLS was increased by 12.9% (Fig 1C) and 2.9% (S1D Fig). There are contradictory reports about the effect of RU on the lifespan of adult flies. A recent study on this topic revealed a positive impact on female flies highly depending on the genotype and the applied RU concentration [20], whereas other studies did not report any effect of RU [21]. However, only RU concentrations equal or higher than 10 μg/ml have been shown to effect lifespan of some genotypes, which is 10 to 20 fold higher than the concentrations we applied to induce *Dro* or *CecA1* expression in *Tub*^*GS*^ flies. Additionally, we could not induce any changes of the lifespan by feeding RU to *Tub*^*GS*^ or *white* flies at concentrations of 0.5 or 1 μg/ml (S1E and S1F Fig).

Modulation of *Drosophila* gut immune functions has been linked to ageing in several previous studies [9,15]. In *qPCR* analysis of *Dro* or *CecA1* levels in midguts of RU treated *Tub*^*GS*^*>Dro* or *Tub*^*GS*^*>CecA1* flies, respectively, we measured significantly induced expression (Fig 1D and 1E). However, the induction of AMP gene expression in these flies is not limited to the intestinal tract. In order to investigate if AMP induction exclusively in the gut is able to increase the lifespan of *Drosophila*, we used the RU inducible midgut-specific *Ti*^*GS2*^*-gal4* driver (*Ti*^*GS2*^) [22] for subsequent experiments. Due to the fact that we obtained the maximal and most stable lifespan extension by overexpressing *Dro* (Fig 1B; S1A and S1B Fig) we focused on this AMP. Using 10 μg/ml RU led to a significant *Dro* transcript induction in midguts (Fig 1F). We could observe a significant increased MLS of 7.7%, 2.8%, 3.6% and 7.4% in four independent experiments (Fig 1G; S1G–S1I Fig). Applying a lower concentration of 1 μg/ml RU was not sufficient to induce a lifespan effect (S1J Fig). An impact on lifespan by RU itself was excluded by feeding 10 μg/ml RU to *Ti*^*GS2*^ and *white* control flies (S1K and S1L Fig). Altogether these findings demonstrate that the expression of a single AMP in the midgut is sufficient to extend the lifespan of *Drosophila*.

### *Dro* extends lifespan by enhancing intestinal immunity

**Fig 2:**
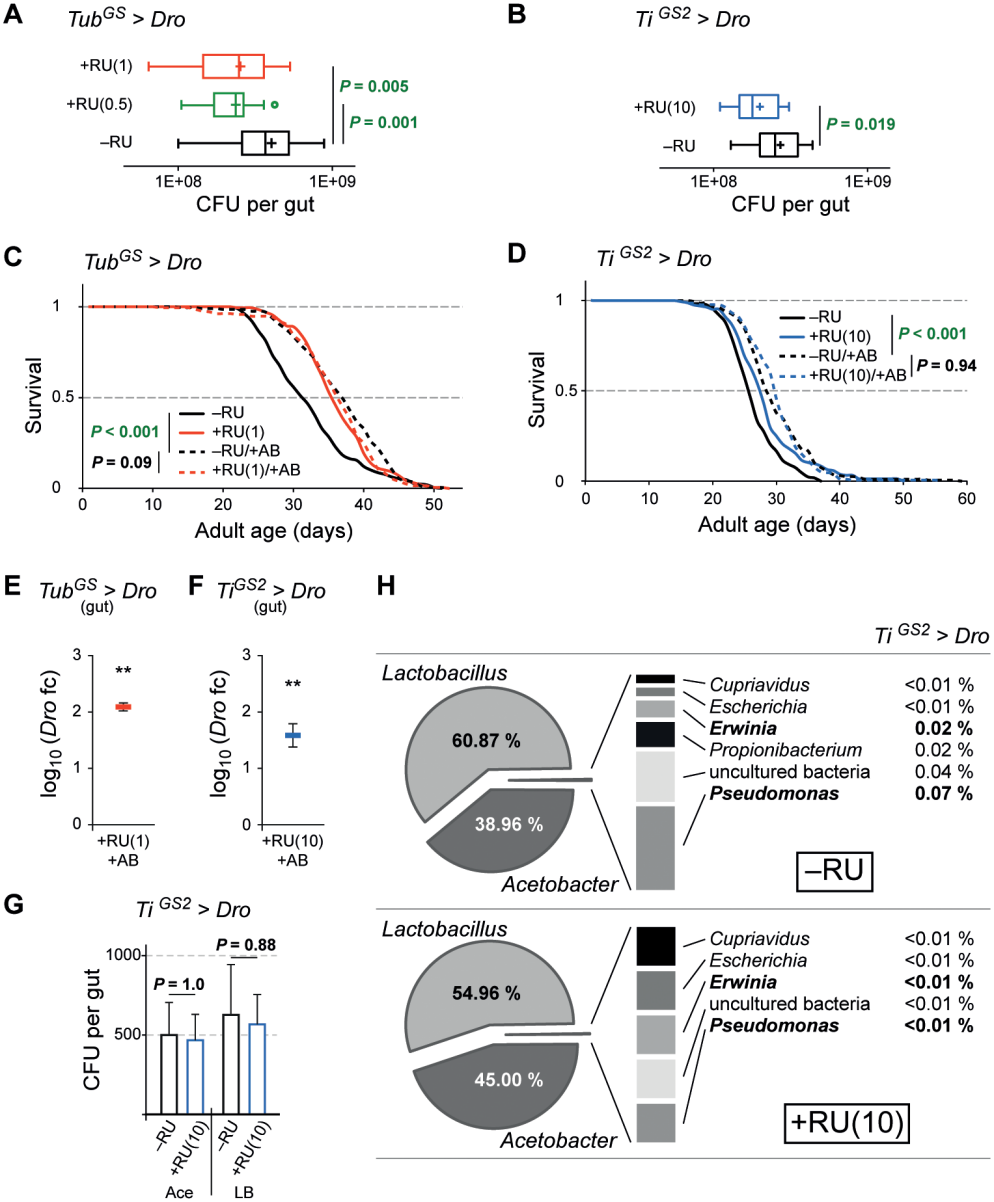
*Dro* enhances intestinal immunity and thereby extends lifespan. (A–B) Ubiquitous or gut-specific expression of *Dro* enhances intestinal immunity against pathogens. Persistence of *Pe* in female *Tub*^*GS*^*>Dro* (A) and *Ti*^*GS2*^*>Dro* (B) flies fed ± 0.5 or 1 μg/ml RU (A), or ± 10 μg/ml RU (B). *n* ≥ 15 replicates of one or two midguts (two biological repeats). (C–D) Flies kept on antibiotic-food live longer. Additional ubiquitous (C) or gut-specific (D) expression of *Dro* has no effect. (C) Lifespan analysis of antibiotic treated *Tub*^*GS*^*>Dro* flies fed ± 1 μg/ml RU (–RU/+AB, dashed black; +RU/+AB, dashed red) and control flies fed ± 1 μg/ml RU (–RU, solid black; +RU solid red). Note that–RU and +RU are the same cohorts as in Fig 1B. *n* ≥ 217 flies/condition. (D) Lifespan analysis of antibiotic treated flies fed ± 10 μg/ml RU (–RU/+AB, dashed black; +RU/+AB, dashed blue) and control flies fed ± 10 μg/ml RU (–RU, solid black; +RU solid blue). Note that –RU and +RU are the same cohorts as in Fig 1G. *n* ≥ 169 flies/condition. (E–F) *Dro* transcription in the midgut of 14-day old female *Tub*^*GS*^*>Dro* (E) and *Ti*^*GS2*^*>Dro* (F) flies kept on antibiotic-food with 1 (E) or 10 (F) μg/ml RU. Mean *log*_*10*_ of fold change is compared to –RU/+AB controls (set to 0, not shown). *n* = 3 (E), *n* = 4 (F) replicates of five midguts. (G) Overexpression of *Dro* has no effect on intestinal microbiota. CFU analysis of 14-day old female *Ti*^*GS2*^*>Dro* flies fed ± 10 μg/ml RU. Midgut homogenates were plated on lysogeny broth medium (LB) or on selective medium for *Acetobacteriaceae* (Ace). *n* = 3 replicates of ten midguts. (H) Microbiota analysis in the midgut of female *Ti*^*GS2*^*>Dro* flies fed ± 10 μg/ml RU on standard food. Statistical tests: (A) Fisher´s LSD test (ANOVA), (B), (G, right side) two-sample t-test, (C–D) log-rank test (Kaplan-Meier analysis), (E–F) one sample t-test, (G, left side) Mann-Whitney test. ** *P* ≤ 0.01. Error bars in (E–G) represent the standard error of the mean. AB, antibiotic treatment; *Dro*, *Drosocin*; *CecA1*, *CecropinA1*; CFU, colony-forming units; +RU, RU treatment. Genotypes see Fig 1.

The activation of the IMD pathway and therefore the induction of a set of AMPs as well as the isolated expression of AMPs have been reported to protect flies against oral infections [10]. To examine whether the constitutive expression of *Dro* has the potential to improve protection against natural pathogen infections we infected *Tub*^*GS*^*>Dro* and *Ti*^*GS2*^*>Dro* flies orally with the natural, food-borne and mainly gut confined *Drosophila* pathogen *Pseudomonas entomophila* (*Pe*) [11]. After four hours of infection we analyzed the *Pe* colony forming units in the gut of the flies. The ubiquitous and also the gut specific expression of *Dro* resulted in a significant reduction of the *Pe* bacterial load in RU treated flies (Fig 2A and 2B). To exclude a direct effect of RU on *Pe* growth, we plated *Pe* cultures on agar plates containing 1, 10 or 100 μg/ml RU. We could not observe any effect of RU on bacterial growth (S2A Fig). These results indicate that an enhanced resistance against food-borne pathogens could contribute to the lifespan extension observed in flies with increased intestinal AMP expression levels.

To investigate this further, we kept *Tub*^*GS*^*>Dro* and *Ti*^*GS2*^*>Dro* flies on antibiotic-food. We applied a mixture of three antibiotics using concentrations, which have previously been shown to effectively inhibit bacterial growth generating a germ-reduced environment and an diminished endogenous bacterial load in the gut [23–25]. These effects have also been described in several other studies using different antibiotics or combinations of antibiotics [9,26,27]. Notably, it has been reported that the antibiotics used in this study do not interfere with IMD pathway activation [24] and that they have no influence on food intake [23,25]. Nevertheless, we cannot completely rule out such effects. We found that in our experimental conditions female *Tub*^*GS*^*>Dro* and *Ti*^*GS2*^*>Dro* flies fed with antibiotics lived significantly longer than untreated control animals. The MLS was increased by 15.6% and 11.5% respectively (Fig 2C and 2D). The lifespan extension of *Tub*^*GS*^*>Dro* and *Ti*^*GS2*^*>Dro* flies by feeding antibiotics was in the same range as by RU-induced *Dro* expression (Fig 2C and 2D). A significantly increased MLS of 7.9% by antibiotic treatment was also observed in a control experiment using *white* flies (S2B Fig). Additional induction of *Dro* in flies being kept on antibiotic-food did not further prolong the lifespan of *Tub*^*GS*^*>Dro* and *Ti*^*GS2*^*>Dro* flies (Fig 2C and 2D). The RU dependent *Dro* induction in the midguts of flies on antibiotic-food was verified by qPCR (Fig 2E and 2F). We speculate that *Drosophila* flies maintained on antibiotic food face a reduced amount of pathogens and therefore, increased levels of *Dro* in the gut do not provide an additional longevity benefit.

We were then interested if we harbor potentially harmful bacteria in flies of our lab, which could be targets for *Dro*. We measured the diversity of the midgut associated microbiota in 14 day-old female *white* flies by 16S rDNA sequencing (S2D Fig) and found two previously described opportunistic *Drosophila* pathogens, namely the Gram-positive *Enterococcus faecalis* [28] and the Gram-negative *Providencia sp*. [29]. Beside the commonly known commensal *Lactobacillus* and *Acetobacter* species, we could additionally identify several unclassified bacterial strains including the Gram-negative *Elizabethkingia menigoseptica*, which has been reported as a natural gut pathogen of *Anopheles gambiae* [30]. Next we tried to obtain insights into qualitative and quantitative changes of the intestinal microbiota upon *Dro* expression. To investigate quantitative changes, we plated dissected midgut homogenates of 14 day-old female *Ti*^*GS2*^*>Dro* flies treated with or without RU on lysogeny broth medium (LB) and selective medium for *Acetobacteriaceae*, which represents one of the major species of the *Drosophila* intestinal microbiota. We could not observe a difference in bacterial growth in both conditions (Fig 2G). To exclude a direct effect of RU on bacterial growth, we plated cultures obtained from *Ti*^*GS2*^*>Dro* midguts on agar plates containing 1, 10 or 100 μg/ml RU. We could not detect a significant effect of RU on bacteria growing on LB or selective *Acetobacteriaceae* plates (S2C Fig). For qualitative analysis of the microbiota, we measured the ratio of the bacterial genera in dissected midguts of 14 day-old female *Ti*^*GS2*^*>Dro* flies with and without RU induction by next generation sequencing (Fig 2H). The midguts of these flies were dominated by *Lactobacillus* and *Acetobacter*, which upon *Dro* expression showed only a small shift of the ratio in favor of *Acetobacter* (Fig 2H). Such a change has not been reported to influence lifespan in *Drosophila*. Interestingly, we found a low amount of the two Gram-negative bacterial genera *Pseudomonas* and *Erwinia*. These genera include well known natural *Drosophila* pathogenic species like *Pe* [11] and *Erwinia carotovora* [31]. Taking together these results, we could not find an evidence of a relevant impact of *Dro* expression on the composition and the amount of the commensal microbiota. On the other hand, finding several potentially pathogenic bacterial species strengthens the hypothesis that the impact of AMPs on pathogens could be responsible for the observed lifespan extension.

### *Dro* expression reduces intestinal stress response, immune and regenerative activity

**Fig 3:**
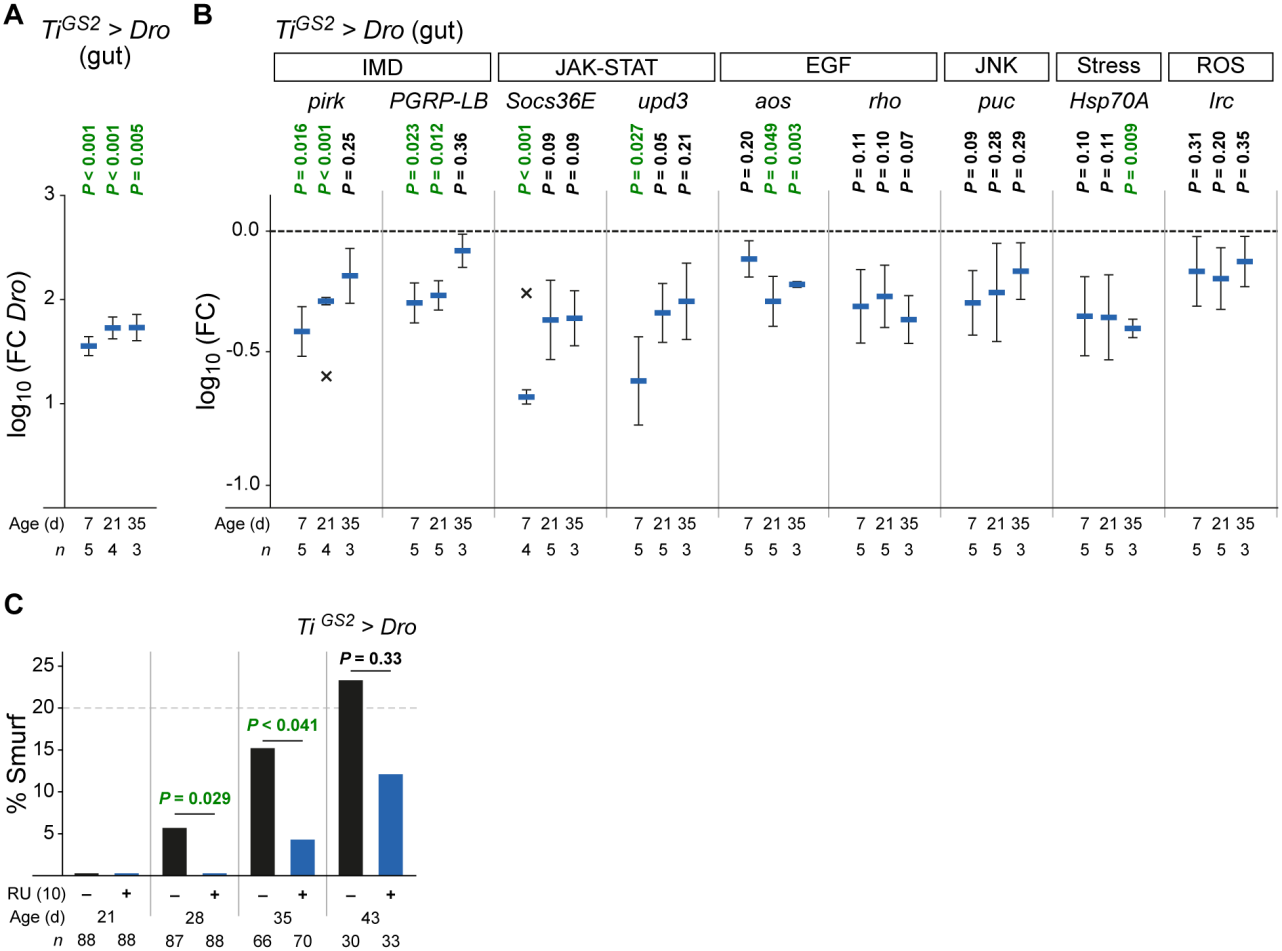
*Dro* expression reduces intestinal stress response, immune and regeneration activity. (A) *Dro* transcription in the gut of female *Ti*^*GS2*^*>Dro* flies fed with 10 μg/ml RU at different ages. (B) Gut-specific expression of *Dro* reduces transcription of genes involved in stress response, immune and regeneration activity in the intestinal tract. Transcriptional analysis of different pathways in midguts of female *Ti*^*GS2*^*>Dro* flies fed with 10 μg/ml RU at different ages. For details of the tested genes and pathways see main text. (A–B) Mean l*og*_*10*_ of fold change is compared to controls of same age fed without RU (set to 0, not shown). *n* = number of replicates of five guts. X represents outliers (Grubbs test, see Materials and methods): –0.57 (*pirk*), –0.24 (*Socs36E*). Note that data in (A) and (B) originate from the same experiments. (C) *Dro* transcription in the gut reduces intestinal damage. Smurf analysis of female *Ti*^*GS2*^*>Dro* flies fed ± 10 μg/ml RU at different ages. *n* = number of flies. Statistical tests: (A–B) one sample t-test, (C) Fisher´s exact test. Error bars represent the standard error of the mean. *aos*, *argos*; *Dro*, *Drosocin*; EGF, Epidermal growth factor signaling; *Hsp70A*, *Heat-shock-protein-70A*; IMD, Immune Deficiency pathway; *Irc*, *Immune-regulated catalase*; JAK-STAT, Janus kinase / Signal Transducer and Activator of Transcription pathway; JNK, c-Jun N-terminal kinase signaling; *PGRP-LB*, *Peptidoglycan recognition protein LB*; *pirk*, *poor Imd response upon knock-in*; *puc*, *puckered*; *rho*, *rhomboid*; ROS, Reactive oxygen species production; *Socs36E*, *Suppressor of cytokine signaling at 36E*; *upd3*, *unpaired 3*. Genotype was: *w/y*,*w;UAS-Dro/+;TiGS2*^*GeneSwitch*^*-gal4/+ (Ti*^*GS2*^*>Dro)*.

The *Drosophila* intestine has been described to be an important mediator of organismal lifespan [32]. It has been shown that gut senescence is linked to organismal ageing in several studies [33–35]. Importantly, the ageing of the gut can be caused by the microbe-induced disruption of gut homeostasis leading to dysplasia and finally to the loss of epithelial barrier integrity [14,15,25]. We wanted to know if the *Dro* expression in the midgut of the fly and its impact on pathogenic or commensal bacteria positively influences the intestinal homeostasis and thereby leads to the observed lifespan increase. The induction by RU led to an increase of *Dro* transcript levels in midguts of female *Ti*^*GS2*^*>Dro* flies throughout lifetime (Fig 3A). In these animals we analyzed the intestinal expression levels of several markers of important pathways linked to intestinal homeostasis, immunity and ageing by qPCR at three different time points during the adult stage (Fig 3B). An expression level decrease of these markers indicates a reduction of their respective signaling pathway. At every time point all marker genes are down regulated, most prominently the ones of the IMD and the JAK-STAT pathways. Both pathways show their strongest reduction in young and middle aged animals. AMP expression in the *Drosophila* midgut is primarily regulated through IMD signaling. The pathway is activated in response to infections [8,31] and by the commensal microbiota [9]. Pirk (Poor IMD response upon knock-in) [36] and PGRP-LB (Peptidoglycan recognition protein LB) [37] are negative regulators of IMD signaling activated by the pathway itself. The JAK-STAT and the EGF (Epidermal growth factor) pathways are necessary to stimulate proliferation and differentiation of intestinal stem cells (ISCs) and thereby maintaining midgut epithelial homeostasis and regeneration [38,39]. Their infection dependent activation has been described to induce the transcription of the JAK-STAT pathway components Socs36E (Suppressor of cytokine signalling at 36E), which is a pathway repressor, and Upd3 (Unpaired 3), a ligand of the Domeless receptor. EGF pathway activation was measured by testing the pathway components Aos (Argos), a positively regulating kinase and the negative regulator Rho (Rhomboid) which are transcriptionally regulated [8,14,40]. We also tested the activity of the cytoprotective JNK (c-Jun NH2 terminal kinase) signaling pathway, which has been shown to be required for midgut regeneration [33]. The pathway is activated upon infection, monitored by the transcriptional activation of the target gene *puc* (*puckered*) [8,14]. Another infection-induced transcriptionally regulated gene is the stress response factor *Hsp70A* (*Heat shock protein 70A*) [8]. Finally, *Irc* (*Immune-regulated catalase*) is a marker for the activation of the reactive oxygen species (ROS) pathway and is essential for ROS detoxification [41].

To verify the reported pathogen dependent activation of these pathways [8] we infected uninduced *Ti*^*GS2*^*>Dro* flies with *Pe* and measured the transcriptional regulation two and 16 hours after infection. All target genes showed dramatically increased transcription levels providing additional evidence for the infection-induced activation of the tested pathways (S3A Fig). To exclude an influence of the inducer on the target gene transcription levels we fed 10 μg/ml RU to *white* flies, which did not result in a change of expression (S3B Fig). Taken together the expression levels of the tested target genes are decreased upon *Dro* induction, contrary to the observed increase due to the *Pe* infection.

To examine if these observed alterations in pathway activity impact the physiology of the gut we performed an intestinal integrity assay which is a marker for intestinal aging [16,35]. Every 7 days we fed flies the non-absorbable blue dye Erioglaucine that cannot cross an intact gut epithelium. The 'Smurf' phenotype, which means that the blue dye leaches into the hemolymph and spreads in the whole body cavity, was scored. This phenotype reflects the loss of intestinal barrier integrity leading to the death of the fly [16]. In two independent experiments we could not detect any Smurf phenotype differences in 21 days old flies expressing *Dro* compared to control animals. However, we found a significant lower number of middle-aged (28 or 35 days old) Smurf flies (Fig 3C; S3C Fig). In 43 days old flies we could not detect a significant difference in gut integrity anymore. These observations suggest that *Dro* expressing *Ti*^*GS2*^*>Dro* flies experience a reduced immune challenge, less stress and a lowered need for intestinal regeneration activity, which leads to an improved intestinal integrity.

## Discussion

Our data demonstrate that both the ubiquitous as well as the gut specific induction of *Dro* and *CecA1* is sufficient to significantly prolong lifespan of adult flies. These animals show reduced activation of classical immune pathways such as IMD and JAK-STAT over lifetime, less JNK and EGF pathway activity, required for regeneration, stem cell maintenance and a reduced stress response. These pathways usually increase their activity during ageing or upon bacterial challenge and are considered as markers for intestinal homeostasis [13,14]. We could also provide evidence for an improved intestinal barrier integrity which delays intestinal and organismal ageing and finally leads to longer lifespan of flies overexpressing *Dro*. We suggest that the reduction of bacterial challenges upon *Dro* expression is responsible for these effects. Consistently, *Dro* expression was able to combat the infection with the natural *Drosophila* pathogen *Pe* in an oral infection model. Such a protection by single or several AMPs has been reported for some systemic infection models [42] and also for the local protection against *Pe* by expressing *Diptericin* or *Attacin* [10]. Antibiotics diminish the bacterial load of the food and the amount of the commensal as well as the pathogenic bacteria of the fly [26]. Ren et al. reported that neither the treatment with antibiotics nor the subsequent reduction or loss of commensal bacteria do seem to have an effect on the lifetime of male *Drosophila* flies [26]. In contrast, in our experimental conditions using mated female flies, feeding antibiotics significantly prolonged lifespan. This may be explained by a different composition of commensal and pathogenic bacteria contained in our fly strains maintained under our laboratory conditions (see gut microbiota analysis in Fig 2H) or the differing gut physiology between male and female flies. Expression of *Dro* in flies treated with antibiotics did not further prolong the lifespan of these animals. We assume that this is due to the loss of bacterial targets, which *Dro* could act on. It has been reported that an overexpression of multiple as well as single AMPs by constitutive induction or by loss of their negative regulator *caudal* [9] can lead to negative effects on gut health and survival. To our knowledge, the effect of *Dro* expression has not been investigated in any study. Ryu et al. have observed a pathogenic phenotype of AMP expression only under conventional rearing conditions. This phenotype could be rescued by germ free conditions, explaining the observed effects with intestinal dysbiosis and a subsequent rupture of gut homeostasis [9]. Although the study reports a negative effect of AMP induction in the gut, this still supports our hypothesis that AMPs are reliant on bacteria as targets to influence gut homeostasis and organismal lifespan. A shift in the commensal community structure favoring the growth of the usually minor species *Gluconobacter morbifer G707* has been identified to be responsible for the pathogenic phenotype of altered AMP expression [9]. In our study we could not detect a similar severe effect on the quality or the quantity of the microbiota nor could we detect *Gluconobacter*. Taken together, our results do not indicate that an effect of *Dro* on the commensal microbiota is responsible for the longevity of the flies, which we nevertheless do not entirely exclude. On the other hand, we could identify several potentially pathogenic bacteria in our flies, leading us to the assumption that constitutive induction of AMP expression may effectively counteract the first steps of natural pathogen infections, thereby reducing the gastrointestinal infection rates over lifetime. Besides their antimicrobial function, we do not exclude other positive effects of AMPs on lifespan. As an example, in the study of Zhao et al. *Diptericin* has been shown to prolong lifespan by increasing tolerance to oxidant stress [43]. In vertebrates AMPs have been reported to inhibit tumor cell proliferation by targeting the negatively charged cancer cells [44].

AMPs could play an important role to mediate longevity in diverse contexts. As just one example, our previous studies in *Drosophila* larvae have shown that AMPs can be activated infection-independent in response to the metabolic status by dFOXO, a transcriptional regulator of the insulin signaling pathway. Down regulation of insulin signaling leads to dFOXO activation and is associated with longevity in various animal systems [6,21]. Loss and gain-of-function studies in adult flies indicate that *Dro* can be activated by dFOXO in the midgut improving the immune response (unpublished data). This hypothesis is also supported by a recent study showing that dFOXO signaling leads to AMP induction and is required to survive oral infections [45]. Taken together our findings provide new insights into the network of metabolism, innate immunity, homeostasis and ageing, helping to understand the complex relationship of these fields of research.

## Materials and methods

### Fly strains

All stocks were maintained at 25°C on a 12 hour light: 12 hour dark cycle on standard food containing 57 g/l cornmeal (Bedorf Mühle, Wachtberg-Villip, Germany), 11.5 g/l yeast (Gewürzmühle Brecht, Eggenstein-Leopoldshafen, Germany), 6 g/l agar-agar (Gewürzmühle Brecht), 7% sugar beet molasses (Grafschafter Krautfabrik, Meckenheim, Germany) and 1.4 g/l Nipagin (Sigma-Aldrich, St. Louis, MO). All experiments have been performed with female flies. RU inducible *gal4* expressing lines were *Tubulin-GeneSwitch* (*Tub*^*GS*^*-gal4*) [19] and *Ti-GeneSwitch* (*Ti*^*GS2*^*-gal4*) [22]. For the overexpression of AMPs we used the following fly strains: *UAS-Dro/CyO* (*UAS-Dro*) and *UAS-CecA1* (*UAS-CecA1*) [42]. The *white* strain used is *white*^*1118*^ from Bloomington stock center (line #5905).

### Lifespan analysis

For longevity analysis flies were collected within 24 hours, transferred to fresh vials and left to mate for 48 hours. Female flies were then randomly sorted into vials at a density of 22 individuals per vial. The recipe for lifespan analysis food was modified from Chapman and Partridge [46]: 7.5% yeast autolysate (Sigma-Aldrich), 7.5% glucose (Roth, Karlsruhe, Germany), 2.1% ethanol (Roth), 2% Kobe I agar (Roth), 0.3% Nipagin (Sigma-Aldrich). To induce geneswitch-gal4 dependent expression, RU486 (Mifepristone, Cayman Chemical Company, Ann Arbor, MI) was added to the food at given concentrations. Antibiotics were applied as a mixture in concentrations of 500 μg/ml ampicillin (Roth), 50 μg/ml tetracycline (Sigma-Aldrich) and 200 μg/ml rifampicin (Sigma-Aldrich) [23]. Flies were transferred to fresh food every two or three days. Deaths were scored every day.

Calculation of survival, mean lifespan, median lifespan, and Log-rank test was done by Kaplan-Meier analysis using MS Excel and XLSTAT. All values for lifespan experiments are given in S2 Table and S1–S4 Files.

### Quantitative *real time* PCR

Four to eight *Drosophila* adults or ten guts were shock frozen in liquid nitrogen and crushed with a micro-pestle. If not stated elsewise two weeks old flies were used. Total RNA was isolated with the NucleoSpin RNA II kit (Macherey & Nagel, Düren, Germany). The kit includes on-column *DNase*I treatment. First strand cDNA reaction was carried out with 500 ng total RNA using QuantiTect Reverse Transcription Kit (Qiagen, Hilden, Germany) including *DNase*I treatment. For quantitative *real time* PCR (qPCR) the reaction consisted of cDNA template (aliquot of cDNA first strand reaction), forward and reverse primers (final concentration 200 nM) and iQ SYBR Green Supermix (BIO-RAD, Munich, Germany) in a total volume of 25 μl. For each template at least two reactions were analyzed in parallel. The experiments were carried out with the CFX96 *real time* PCR Detection System from BIO-RAD. *Ribosomal protein L32* (*RpL32*) was used as the reference gene. qPCR results were analyzed using BIO-RAD CFX manager software, MS Excel and XLSTAT. For statistical analysis fold changes received from CFX manager were *log*_*10*_ transformed, tested for normal distribution (Shapiro-Wilk test) and outliers (Grubbs test). Outliers were taken out of the analysis and are represented as “X” in Fig 3B (*pirk* and *Socs36E*). Significance was tested by one-sample t-test (for normally distributed data) or Wilcoxon signed-rank test (for not normally distributed data). All qPCR values (*n*, fold change, *log*_*10*_, SEM, *P*-value, etc.) are given in S1 Table.

The following oligonucleotides were tested for efficiency and used for qPCR analysis: *aos*: CAA CAG CAG CAT CGC A (Dm-Aos-sy-F1), ACA GAC GGG CAA ATC CT (Dm-Aos-sy-R1); *CecA1*: TCT TCG TTT TCG TCG CTC TCA (CecA1-sy-F1), ATT CCC AGT CCC TGG ATT GTG (CecA1-sy-R1); *Dro*: TTT GTC CAC CAC TCC AAG CAC (Dro-sy-F1), ATG GCA GCT TGA GTC AGG TGA (Dro-sy-R1); *Hsp70A*: CGA TGC CAA GAT GGA TAA GG (Dm-Hsp70ab-sy-F2), CTG GGT TGA TGG ATA GGT TG (Dm-Hsp70ab-sy-R2); *Irc*: AAA GCG ACT GGA GGA CAA TC (Dm-Irc-sy-F1), GAA GTT GAG CGT GTG AAA GG (Dm-Irc-sy-R1); *PGRP-LB*: ATT GAC CCT GCC TAC AAG C (Dm-PGRP-LB-sy-F1), CTT CGG TGT CGT TTA TGT GG (Dm-PGRP-LB-sy-R1); *pirk*: GCG TTC GTG TGA TAG AAA CC (Dm-pirk-sy-F1), GCT CTT ATT GGG GGA TTT ACC (Dm-pirk-sy-R1); *puc*: TCC TTC GTC ATC TTC TGT GG (Dm-Puc-sy-F1), GAC TTG GAT TTA CCC CGT TC (Dm-Puc-sy-R1); *rho*: TTG TCA TCT TTG TCT CCT GC (Dm-Rho-sy-F1), GCA ATG TAC GAC ACC TGG (Dm-Rho-sy-R1); *RpL32*: GCT AAG CTG TCG CAC AAA TG (rp49-Real-F1), GTT CGA TCC GTA ACC GAT GT (rp49-Real-R1); *Socs36E*: CTT TCA ATG GGA GCA GCA AC (Dm-Socs-sy-F1), CGA GGA TGT GGA TGT GGA C (Dm-Socs-sy-R1); *upd3*: CGA AAC CTC CAT TCC ACA C (Dm-Upd3-sy-F1), ATT CCT CGT GAT TTG TCG TG (Dm-Upd3-sy-R1).

### Infection and bacterial persistence assays

For infections we used *Pseudomonas entomophila* carrying a rifampicin resistance [11]. The bacteria were cultured in lysogeny broth (LB) medium (Roth) with rifampicin (100 μg/ml) at 30°C. For oral infection experiments and bacterial persistence assays we used modified protocols previously described by Vodovar and colleagues [11]. A group of female adult flies at the age of two weeks was placed on a filter soaked with infection solution. For this infection solution a pellet of a 24 hour culture of bacteria was mixed with 5% sucrose/phosphate buffered saline (PBS, Roth) in the ratio 1: 4. For the bacterial load assay the flies were infected at 25°C for four hours. The flies were externally disinfected in 70% ethanol and individually homogenized in 10 mM MgSO_4_ (Roth) with glass beads (Roth), using a Precellys homogenizer (VWR, Radnor, PA) for 10 seconds and 5000 rpm. The homogenate was spread onto LB plates containing rifampicin (100 μg/ml) and colonies were counted after 24 hours on 30°C. The CFU count values for each experimental condition were combined, box plotted and analyzed with the Shapiro-Wilk test for normal distribution and the Fisher´s LSD test (ANOVA) (Fig 2A) or the two sample t-test (Fig 2B) for significance using MS Excel and XLSTAT. In box plot diagrams, lines and boxes represent the median and first and third quartiles of the values. Crosses display the mean values. Whiskers extend to upper and lower limits representing values within the 1.5-fold interquartile range. Values outside the 1.5-fold interquartile range are defined as outliers (circles). For controls dilution series of cultured *Pe* have been plated on LB medium containing 1, 10 or 100 μg/ml mifepristone. For controls relative CFU count values were calculated. L*og*_*10*_ transformed relative CFU values were analyzed by Shapiro-Wilk test for normal distribution and the one-sample t-test for significance using MS Excel and XLSTAT. All values (*n*, CFU, SEM, *P*-value, etc.) are given in S3 Table.

### Quantification of bacteria from *Drosophila* midgut

Midgut bacteria of 14-day old female *Ti*^*GS2*^*>Dro* flies fed with or without 10 μg/ml RU have been isolated as described in the "Isolation and identification of bacteria from *Drosophila* midgut" section (see below). Dilution series of isolated bacteria have then been plated on lysogeny broth medium containing 25 g/l LB broth, 15 g/l Kobe I agar or on selective plates for *Acetobacteriacea* containing 25 g/l D-mannitol (Roth), 5 g/l yeast extract (Roth), 3 g/l peptone (Roth), 15 g/l Kobe I agar (Roth) [15]. The plates have been cultured at 30°C for 48 h and afterwards counted for colony forming units (CFUs). The CFU count values were analyzed with the Shapiro-Wilk test for normal distribution and the two-sample t-test (normal distribution) or the Mann-Whitney test (no normal distribution) for significance using MS Excel and XLSTAT. For controls dilution series of isolated bacteria have been plated on LB medium or selective medium containing 1, 10 or 100 μg/ml mifepristone. For controls relative CFU count values were calculated. L*og*_*10*_ transformed relative CFU values were analyzed by Shapiro-Wilk test for normal distribution and the one-sample t-test (normal distribution) or the Wilcoxon signed-rank test (no normal distribution) for significance using MS Excel and XLSTAT. All values (*n*, CFU, SEM, *P*-value, etc.) are given in S3 Table.

### Isolation and identification of bacteria from *Drosophila* midgut

We used 14 day-old female *white* flies raised on standard food for the isolation of gut bacteria. Prior to gut preparation, the flies were sterilized in 80% ethanol for five minutes to decontaminate any bacterial contaminants on the surface of the flies. Midguts including the proventriculus from a total of five female flies were homogenized with glass beads in 600 μl 1 x PBS and the supernatant containing gut bacteria was transferred to a new 1.5 ml centrifuge tube. The supernatant was centrifuged at 12,000 x g for five minutes to pellet the gut bacteria. Centrifuged cells were resuspended in 100 μl of 1 x PBS and were plated and cultured onto mannitol (25 g/l D-mannitol, 5 g/l yeast extract, 3 g/l peptone, 15 g/l Kobe I agar) or MRS agar containing 55 g/l MRS Broth (Roth), 15 g/l Kobe I agar under aerobic or anaerobic conditions at 30°C for 24 to 48 hours. Gut bacteria were selected based on colony morphology and species were identified by PCR amplification targeting the 16S rDNA gene using modified 27F (AKW GTT TGA TCM TGG CTC AG) and 1492R (GGH TAC CTT GTT ACG ACT T) eubacteria 16S rDNA universal primers. PCR was conducted based on the protocol for the PrimeSTAR HS DNA Polymerase (TakaraBio, Shiga, Japan) with annealing temperature set at 50°C. Sequences of all attained 16S rDNA amplicons were determined by standard Sanger sequencing.

### Gut barrier analysis (Smurf assay)

The integrity of the gut barrier was tested by placing flies on blue dyed lifespan analysis food prepared with 2.5% (w/v) Erioglaucine (Sigma-Aldrich), also known as FD&C blue no 1. Female flies were set up as described in the lifespan analysis section. Flies were kept on blue food for 24 h and the Smurf phenotype was scored for another 24 h. Two independent experiments were carried out. The Smurf assay has been described previously in recent publications [25,47]. Smurf data were analyzed with Fisher´s exact test for significance using MS Excel and XLSTAT. All values are given in S4 Table.

## Supporting information

S1 Fig Expression of AMPs extends lifespan. (PDF)

S2 Fig RU treatment does not impair bacterial growth, antibiotic treatment extends lifespan. (PDF)

S3 Fig Control *qPCR* experiments for *Pe* infection and RU treatment, additional Smurf assay. (PDF)

S1 Table *qPCR* data. (PDF)

S2 Table Lifespan analysis data. (PDF)

S3 Table CFU analysis data. (PDF)

S4 Table Smurf analysis data. (PDF)

S1 File Kaplan-Meier analysis of lifespan data in Fig 1. (XLSX)

S2 File Kaplan-Meier analysis of lifespan data in Fig 2. (XLSX)

S3 File Kaplan-Meier analysis of lifespan data in S1 Fig. (XLSX)

S4 File Kaplan-Meier analysis of lifespan data in S2 Fig. (XLSX)
