# Supplementary material for: Antimicrobial peptides extend lifespan in Drosophila
Source: PLoS One. 2017 May 17;12(5):e0176689. doi: 10.1371/journal.pone.0176689 (PMC5435158; doi:10.1371/journal.pone.0176689)
Supplement: S1 Fig — (PDF) [file pone.0176689.s001.pdf]

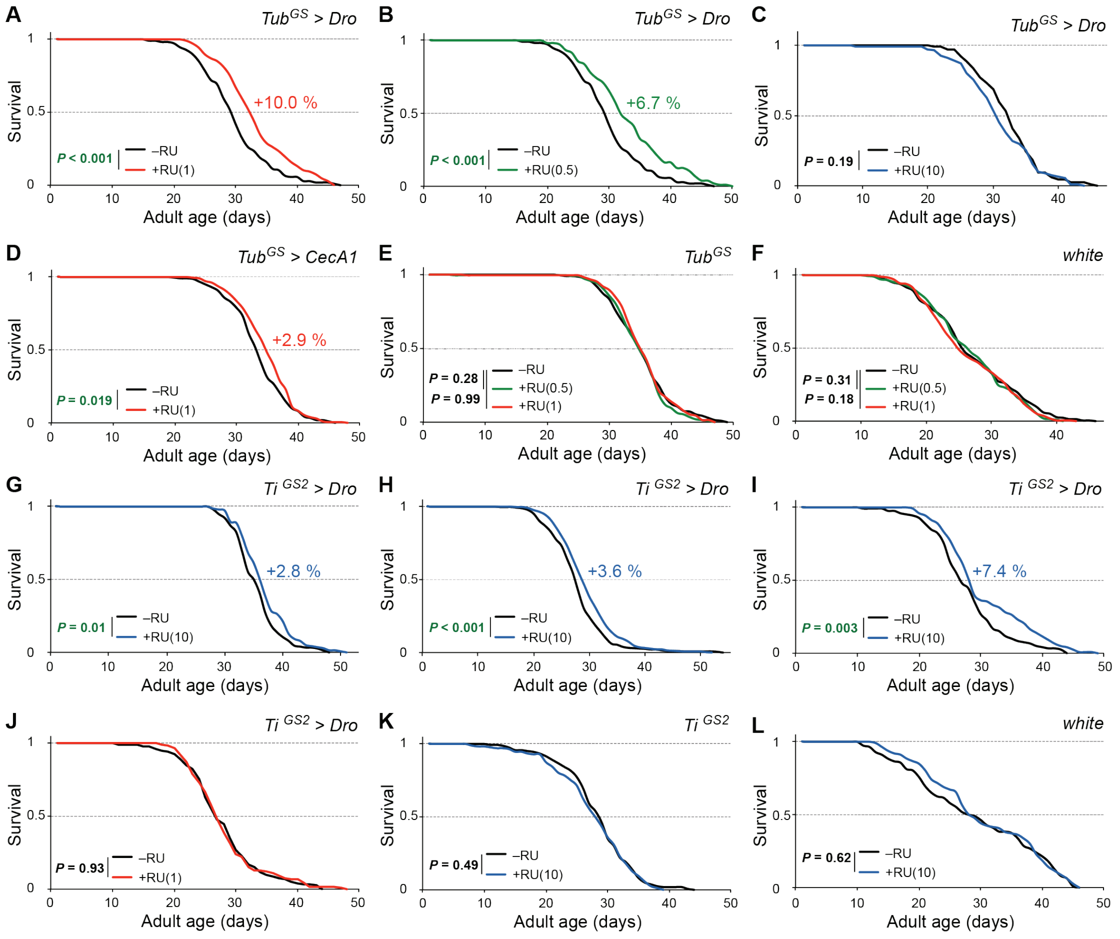

S1 Fig. Expression of AMPs extends lifespan. RU treatment does not extend lifespan. (A–C) Ubiquitous expression of *Dro* extends lifespan. Independent lifespan experiments of female Tub^{GS}>*Dro* flies fed with different concentrations of RU. Compared to controls (black), flies fed with (A) 1 (red) or (B) 0.5 (green) µg/ml RU show 10.0% or 6.7% increased MLS, respectively. (C) Flies fed with 10 (blue) µg/ml RU do not show a significantly increased MLS. Note that controls in (A) and (B) are the same cohort. (D) Ubiquitous expression of *CecA1* extends lifespan. Compared to controls (black) female Tub^{GS}>*CecA1* flies fed with 1 µg/ml RU (red) show 2.9% increased MLS. (E–F) RU treatment does not extend lifespan. Female Tub^{GS} (E) and *white* (F) flies fed with 0.5 (green) or 1 (red) µg/ml RU do not show increased MLS. (G-I) Gut-specific expression of *Dro* extends lifespan. Independent lifespan experiments of female Ti^{GS2}>*Dro* flies fed with 10 µg/ml RU (blue). Compared to controls (black) RU-treated flies show 2.8% (G), 3.6% (H), or 7.4 % (I) increased MLS, respectively. (J) Ti^{GS2}>*Dro* flies fed with 1 µg/ml RU (red) do not show a significantly increased MLS. (K–L) RU treatment does not extend lifespan. Female

Ti^{GS2} (K) and *white* (L) flies fed with 10 µg/ml RU (blue) do not show increased MLS. Statistical tests: log-rank test (Kaplan-Meier analysis). For *n*-, *P*-values and raw data see S2 Table and S3 File. *CecA1*, *Cecropin A1*; *Dro*, *Drosocin*; MLS, median lifespan; +RU, RU treatment. Genotypes were:

w/y,w;UAS-Dro/+; tubulin^{GeneSwitch}*-gal4/+* (*Tub*^{GS}*>Dro*),

w/y,w;+/+;tubulin^{GeneSwitch}*-gal4/UAS-CecA1* (*Tub*^{GS}*>CecA1*),

w/y,w;+/+; tubulin^{GeneSwitch}*-gal4/+* (*Tub*^{GS}),

w/y,w;UAS-Dro/+; TiGS2^{GeneSwitch}*-gal4/+ (Ti*^{GS2}*>Dro),*

y,w/y,w;+/+;TiGS2^{GeneSwitch}*-gal4/TiGS2*^{GeneSwitch}*-gal4 (Ti*^{GS2}),

w/w;+/+;+/+ (*white*).
