# Supplementary material for: Antimicrobial peptides extend lifespan in Drosophila
Source: PLoS One. 2017 May 17;12(5):e0176689. doi: 10.1371/journal.pone.0176689 (PMC5435158; doi:10.1371/journal.pone.0176689)
Supplement: S2 Fig — (PDF) [file pone.0176689.s002.pdf]

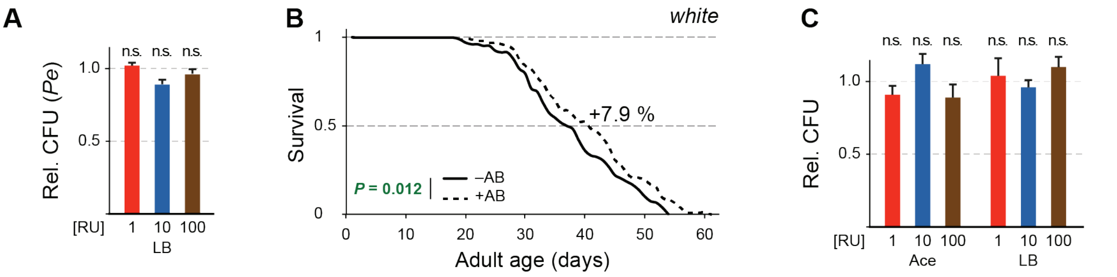

D

Classification	Bacterial isolates	Closest relative (Accession Number)	Similarity (%)
Commensals	*Acetobacter aceti* str. lw-1	*Acetobacter aceti* str. LMG 1531 (AJ419840)	99.9
	Acetobacter cerevisiae str. lw-1	*Acetobacter cerevisiae* str. LMG 1625 (NR_025512)	100.0
	Acetobacter malorum str. lw-1	*Acetobacter malorum* str. LMG 1746 (NR_025513)	99.9
	Acetobacter persici str. lw-1	*Acetobacter persici* str. T-120 (NR_113547)	100.0
	Acetobacter pomorum str. lw-1	*Acetobacter pomorum* str. EW816 (EU096229)	99.9
	Lactobacillus brevis str. lw-1	*Lactobacillus brevis* str. JCM 7775 (AB911501)	99.9
	Lactobacillus fructivorans str. lw-1	*Lactobacillus fructivorans* str. NBRC 14747 (AB680654)	99.9
	Lactobacillus plantarum str. lw-1	*Lactobacillus plantarum* subsp. plantarum ST-III (CP002222)	100.0
Potential Pathogens	*Enterococcus faecalis* str. lw-1	*Enterococcus faecalis* strain WVS464 (KM257719)	100.0
	Providencia sp. str. lw-1	*Providencia vermicola* strain CGS6 (KF886276)	100.0
	Elizabethkingia sp. str. lw-1	*Elizabethkingia meningoseptica* str. Che01 (JX067927)	99.7
Unclassified	*Cupriavidus* sp. str. lw-1	*Cupriavidus* sp. AP1(2010) (GU566329)	99.9
	Massilia sp. clone str. lw-1	Uncultured *Massilia* sp. clone SL25 (HQ264085)	99.7
	Moraxella sp. str. lw-1	*Moraxella osloensis* strain S44-4 (HM217989)	99.7
	Uncultured bacterium str. lw-1	Uncultured bacterium clone 1H3C_38 (JN229875)	100.0

S2 Fig. RU treatment does not impair bacterial growth, antibiotic treatment extends lifespan. (A,C) Bacterial growth is not altered by RU treatment. Colony forming units (CFU) of cultured *Pe* (A) or cultured microbiota from female fly midguts (C) were analyzed. Cultures were plated on lysogeny broth medium (LB) containing different RU concentrations (1 µg/ml, red; 10 µg/ml, blue; 100 µg/ml, brown). Cultured gut microbiota were additionally plated on selective medium for *Acetobacteriaceae* (Ace). Each condition is compared to corresponding control plates without RU (set to 1, not shown). (B) Female *white* flies kept on antibiotic-food live longer. Compared to controls (–AB), antibiotic-treated flies (+AB, dashed line) show 7.9% increased MLS. (D) Identification and classification of bacteria isolated from midguts of female *white* flies. The nomenclature of bacterial species identified in this work was defined based on the closest relative. Comparison of 16S rDNA sequences between bacterial isolates was conducted using near full-length 16S rDNA sequences. Statistical tests: (A, C) one sample t-tests, except (C, RU10, LB), Wilcoxon signed-rank test, (B) log-rank test (Kaplan-Meier analysis). n.s., $P > 0.05$. Error bars represent the standard error of the mean. +AB, antibiotic treatment; –AB, controls fed without antibiotics; MLS, median lifespan; *Pe*, *Pseudomonas entomophila*. For SEM-, *n*-, *P*-values and raw data see S2 and S3 Tables and S4 File.

Genotype was: *w/w;+/+;+/+* (*white*).
