# Supplementary material for: Antimicrobial peptides extend lifespan in Drosophila
Source: PLoS One. 2017 May 17;12(5):e0176689. doi: 10.1371/journal.pone.0176689 (PMC5435158; doi:10.1371/journal.pone.0176689)
Supplement: S3 Fig — (PDF) [file pone.0176689.s003.pdf]

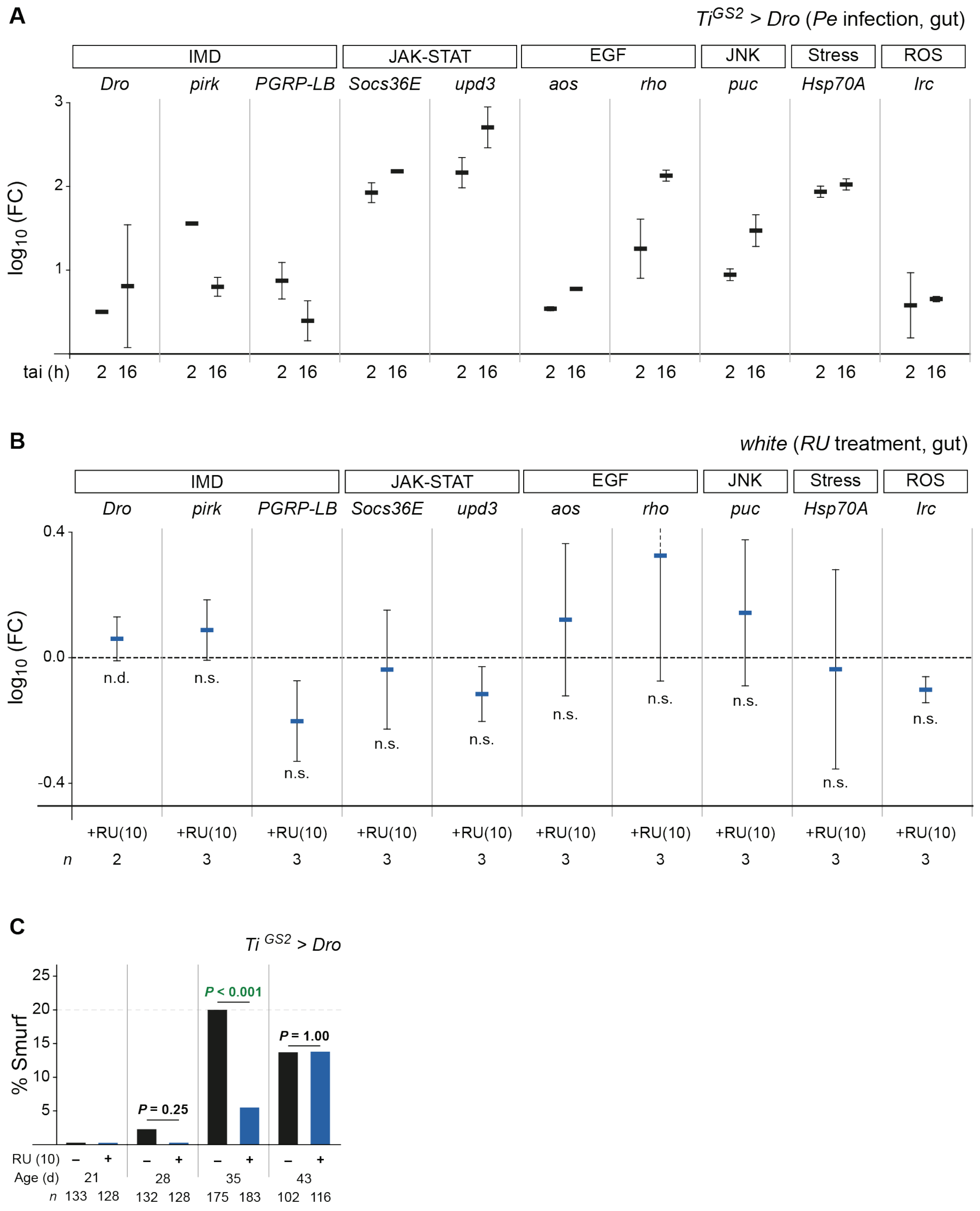

S3 Fig. Control *qPCR* experiments for *Pe* infection and RU treatment, additional Smurf assay. (A) Natural *Pe* infection induces transcription of genes involved in stress response, immune and regeneration activity of the intestinal tract. Transcriptional analysis of different pathways in midguts of female Ti^{GS2}>*Dro* flies infected for two or 16 hours. (B) RU treatment (with 10 µg/ml) has no effect on transcription of genes involved in stress response, immune and regeneration activity in the intestinal tract of female *white* flies. Details to the tested genes and pathways see in the main text. (C) *Dro* transcription in the gut reduces intestinal damage. Smurf

analysis of female Ti^{GS2}>*Dro* flies fed ± 10 µg/ml RU at different ages. *n* = number of flies. Statistical tests: (A) not tested, (B) one sample t-tests (except *Dro*) (C) Fisher′s exact test. n.s., $P > 0.05$; n.d. not tested. Error bars represent the standard error of the mean. For SEM-, *n*-, *P*-values see S1 and S4 Tables. *aos*, *argos*; *Dro*, *Drosocin*; EGF, Epidermal growth factor signaling; *Hsp70A*, *Heat-shock-protein-70A*; IMD, Immune Deficiency pathway; *Irc*, *Immune-regulated catalase*; JAK-STAT, Janus kinase / Signal Transducer and Activator of Transcription pathway; JNK, c-Jun N-terminal kinase signaling; *Pe*, *Pseudomonas entomophila*; *PGRP-LB*, *Peptidoglycan recognition protein LB*; *pirk*, *poor Imd response upon knock-in*; *puc*, *puckered*; *rho*, *rhomboid*; ROS, Reactive oxygen species production; +RU, RU treatment (µg/ml); *Socs36E*, *Suppressor of cytokine signaling at 36E*; tai (h), time after infection (in hours); *upd3*, *unpaired 3*.

Genotypes were:

w/y,w;UAS-Dro/+; TiGS2^{GeneSwitch}*-gal4/+ (Ti*^{GS2}*>Dro)*,

w/w;+/+;+/+ (*white*).
