# Supplementary material for: Antimicrobial peptides extend lifespan in Drosophila
Source: PLoS One. 2017 May 17;12(5):e0176689. doi: 10.1371/journal.pone.0176689 (PMC5435158; doi:10.1371/journal.pone.0176689)
Supplement: S1 Table — (PDF) [file pone.0176689.s004.pdf]

S1 Table. *qPCR* data.

Figure	Genotype	Gene	Probe	Expression (fold change [a])	log_{10} (fold change)	SEM (log_{10})	Normal distribution [b]	*P*-value [c]
**1A**	*Tub*^{GS}*>Dro*	*Dro* (*n* = 3)	–RU	1.0				
			+RU (1)	71.1	1.85	0.37	yes (0.55)	***0.037***
			+RU (10)	241.0	2.38	0.11	yes (0.57)	***0.002***
**1D**	*Tub*^{GS}*>Dro*	*Dro* (*n* = 4)	gut, –RU	1.0				
			gut, +RU (1)	175.3	2.24	0.10	yes (0.87)	***< 0.001***
**1E**	*Tub*^{GS}*>CecA1*	*CecA1* (*n* = 5)	gut, –RU	1.0				
			gut, +RU (1)	86.5	1.94	0.18	yes (0.36)	***< 0.001***
**1F**	*Ti*^{G2S}*>Dro*	*Dro* (*n* = 4)	gut, –RU	1.0				
			gut, +RU (10)	44.0	1.64	0.20	yes (0.14)	***0.004***
**2E**	*Tub*^{GS}*>Dro*	*Dro* (*n* = 3)	gut, +RU (1), –AB	1.0				
			gut, +RU (1), +AB	122.6	2.09	0.07	yes (0.85)	***0.001***
**2F**	*Ti*^{G2S}*>Dro*	*Dro* (*n* = 4)	gut, +RU (1), –AB	1.0				
			gut, +RU (1), +AB	61.8	1.79	0.21	yes (0.22)	***0.003***
**3A**	*Ti*^{G2S}*>Dro*	*Dro* (*n* = 5)	7 d, gut, –RU	1.0				
			7 d, gut, +RU (10)	39.6	1.60	0.09	yes (0.59)	***< 0.001***
		Dro (*n* = 5)	21 d, gut, –RU	1.0				
			21 d, gut, +RU (10)	59.3	1.77	0.11	yes (0.38)	***< 0.001***
		Dro (*n* = 3)	35 d, gut, –RU	1.0				
			33 d, gut, +RU (10)	59.8	1.78	0.13	yes (0.85)	***0.005***
**3B**	*Ti*^{G2S}*>Dro*	*pirk* (*n* = 5)	7 d, gut, –RU	1.0				
			7 d, gut, +RU (10)	–2.5	–0.39	0.10	yes (0.45)	***0.016***
		pirk (*n* = 4)	21 d, gut, –RU	1.0				
			21 d, gut, +RU (10)	–1.9	–0.28	0.01	yes (0.76)	***< 0.001***
		pirk (*n* = 3)	35 d, gut, –RU	1.0				
			33 d, gut, +RU (10)	–1.5	–0.18	0.11	yes (0.40)	***0.246***

S1 Table. *qPCR* data (Continued).

Figure	Genotype	Gene	Probe	Expression (fold change [a])	log_{10} (fold change)	SEM (log_{10})	Normal distribution [b]	*P*-value [c]
		PBRP-LB	7 d, gut, –RU	1.0				
		(*n* = 5)	7 d, gut, +RU (10)	–1.9	–0.28	0.08	yes (0.57)	***0.023***
		PBRP-LB	21 d, gut, –RU	1.0				
		(*n* = 5)	21 d, gut, +RU (10)	–1.8	–0.25	0.06	yes (0.73)	***0.012***
		PBRP-LB	35 d, gut, –RU	1.0				
		(*n* = 3)	33 d, gut, +RU (10)	–1.2	–0.08	0.07	yes (0.55)	***0.360***
		Socs36E	7 d, gut, –RU	1.0				
		(*n* = 4)	7 d, gut, +RU (10)	–4.5	–0.65	0.03	yes (0.65)	***< 0.001***
		Socs36E	21 d, gut, –RU	1.0				
		(*n* = 5)	21 d, gut, +RU (10)	–2.2	–0.35	0.16	yes (0.68)	***0.089***
		Socs36E	35 d, gut, –RU	1.0				
**3B**	*Ti*^{G2S}*>Dro*	(*n* = 3)	33 d, gut, +RU (10)	–2.2	–0.34	0.11	yes (0.94)	***0.087***
		upd3	7 d, gut, –RU	1.0				
		(*n* = 5)	7 d, gut, +RU (10)	–3.9	–0.59	0.17	yes (0.99)	***0.027***
		upd3	21 d, gut, –RU	1.0				
		(*n* = 5)	21 d, gut, +RU (10)	–2.1	–0.32	0.12	yes (0.36)	***0.050***
		upd3	35 d, gut, –RU	1.0				
		(*n* = 3)	33 d, gut, +RU (10)	–1.9	–0.28	0.15	yes (0.42)	***0.208***
		aos	7 d, gut, –RU	1.0				
		(*n* = 5)	7 d, gut, +RU (10)	–1.3	–0.11	0.07	yes (0.45)	***0.202***
		aos	21 d, gut, –RU	1.0				
		(*n* = 5)	21 d, gut, +RU (10)	–1.9	–0.28	0.10	yes (0.77)	***0.049***
		aos	35 d, gut, –RU	1.0				
		(*n* = 3)	33 d, gut, +RU (10)	–1.6	–0.21	0.01	yes (0.41)	***0.003***

S1 Table. *qPCR* data (Continued).

Figure	Genotype	Gene	Probe	Expression (fold change [a])	log_{10} (fold change)	SEM (log_{10})	Normal distribution [b]	*P*-value [c]
3B	Ti^{G2S}>*Dro*	*rho*	7 d, gut, –RU	1.0				
		(*n* = 5)	7 d, gut, +RU (10)	–2.0	–0.30	0.14	yes (0.10)	***0.111***
		rho	21 d, gut, –RU	1.0				
		(*n* = 5)	21 d, gut, +RU (10)	–1.8	–0.26	0.12	yes (0.90)	***0.105***
		rho	35 d, gut, –RU	1.0				
		(*n* = 3)	33 d, gut, +RU (10)	–2.2	–0.35	0.09	yes (0.41)	***0.066***
		puc	7 d, gut, –RU	1.0				
		(*n* = 5)	7 d, gut, +RU (10)	–1.9	–0.28	0.13	yes (0.39)	***0.090***
		puc	21 d, gut, –RU	1.0				
		(*n* = 5)	21 d, gut, +RU (10)	–1.7	–0.24	0.19	yes (0.22)	***0.281***
		puc	35 d, gut, –RU	1.0				
		(*n* = 5)	33 d, gut, +RU (10)	–1.4	–0.16	0.11	yes (0.98)	***0.295***
		Hsp70A	7 d, gut, –RU	1.0				
		(*n* = 5)	7 d, gut, +RU (10)	–2.2	–0.33	0.16	yes (0.75)	***0.098***
		Hsp70A	21 d, gut, –RU	1.0				
		(*n* = 5)	21 d, gut, +RU (10)	–2.2	–0.34	0.17	yes (0.14)	***0.113***
		Hsp70A	35 d, gut, –RU	1.0				
		(*n* = 3)	33 d, gut, +RU (10)	–2.4	–0.38	0.04	yes (0.13)	***0.009***
		Irc	7 d, gut, –RU	1.0				
		(*n* = 5)	7 d, gut, +RU (10)	–1.4	–0.16	0.14	yes (0.20)	***0.314***
		Irc	21 d, gut, –RU	1.0				
		(*n* = 5)	21 d, gut, +RU (10)	–1.5	–0.19	0.12	yes (0.64)	***0.199***
		Irc	35 d, gut, –RU	1.0				
		(*n* = 3)	33 d, gut, +RU (10)	–1.3	–0.12	0.10	yes (0.32)	***0.352***

S1 Table. *qPCR* data (Continued).

Figure	Genotype	Gene	Probe	Expression (fold change [a])	$\log_{10}$ (fold change)	SEM ($\log_{10}$)	Normal distribution [b]	*P*-value [c]
**S1A**	*Ti*^{G2S}>*Dro*	*Dro* (*n* = 2)	2 h pi, gut, –*Pe*	1.0				
			2 h pi, gut, + *Pe*	3.2	0.50	0.02	nd	nd
			16 h pi, gut, –*Pe*	1.0				
			16 h pi, gut, + *Pe*	6.4	0.81	0.73	nd	nd
		pirk (*n* = 2)	2 h pi, gut, –*Pe*	1.0				
			2 h pi, gut, + *Pe*	36.0	1.56	0.004	nd	nd
			16 h pi, gut, –*Pe*	1.0				
			16 h pi, gut, + *Pe*	6.3	0.80	0.11	nd	nd
		PGRP-LB (*n* = 2)	2 h pi, gut, –*Pe*	1.0				
			2 h pi, gut, + *Pe*	7.5	0.87	0.22	nd	nd
			16 h pi, gut, –*Pe*	1.0				
			16 h pi, gut, + *Pe*	2.5	0.40	0.24	nd	nd
		Socs36E (*n* = 2)	2 h pi, gut, –*Pe*	1.0				
			2 h pi, gut, + *Pe*	84.2	1.93	0.12	nd	nd
			16 h pi, gut, –*Pe*	1.0				
			16 h pi, gut, + *Pe*	151.6	2.18	0.003	nd	nd
		upd3 (*n* = 2)	2 h pi, gut, –*Pe*	1.0				
			2 h pi, gut, + *Pe*	145.8	2.16	0.18	nd	nd
			16 h pi, gut, –*Pe*	1.0				
			16 h pi, gut, + *Pe*	507.4	2.71	0.24	nd	nd
		aos (*n* = 2)	2 h pi, gut, –*Pe*	1.0				
			2 h pi, gut, + *Pe*	3.5	0.54	0.02	nd	nd
			16 h pi, gut, –*Pe*	1.0				
			16 h pi, gut, + *Pe*	6.0	0.78	0.01	nd	nd
		rho (*n* = 2)	2 h pi, gut, –*Pe*	1.0				
			2 h pi, gut, + *Pe*	18.0	1.26	0.35	nd	nd
			16 h pi, gut, –*Pe*	1.0				
			16 h pi, gut, + *Pe*	134.5	2.13	0.07	nd	nd

S1 Table. *qPCR* data (Continued).

Figure	Genotype	Gene	Probe	Expression (fold change [a])	log_{10} (fold change)	SEM (log_{10})	Normal distribution [b]	*P*-value [c]
**S1A**	Ti^{G2S}>*Dro*	*puc* (*n* = 2)	2 h pi, gut, –*Pe*	1.0				
			2 h pi, gut, + *Pe*	8.8	0.94	0.07	nd	nd
			16 h pi, gut, –*Pe*	1.0				
			16 h pi, gut, + *Pe*	29.6	1.47	0.19	nd	nd
		Hsp70A (*n* = 2)	2 h pi, gut, –*Pe*	1.0				
			2 h pi, gut, + *Pe*	86.4	1.94	0.07	nd	nd
			16 h pi, gut, –*Pe*	1.0				
			16 h pi, gut, + *Pe*	105.6	2.02	0.19	nd	nd
		Irc (*n* = 2)	2 h pi, gut, –*Pe*	1.0				
			2 h pi, gut, + *Pe*	3.8	0.58	0.39	nd	nd
			16 h pi, gut, –*Pe*	1.0				
			16 h pi, gut, + *Pe*	4.5	0.66	0.03	nd	nd
**S1B**	*white*	*Dro* (*n* = 2)	gut, –RU	1.0				
			gut, +RU (10)	1.1	0.06	0.07	nd	nd
		pirk (*n* = 3)	gut, –RU	1.0				
			gut, +RU (10)	1.2	0.09	0.10	yes (0.61)	***0.458***
		PGRP-LB (*n* = 3)	gut, –RU	1.0				
			gut, +RU (10)	0.6	–0.20	0.13	yes (0.84)	***0.256***
		Socs36E (*n* = 3)	gut, –RU	1.0				
			gut, +RU (10)	0.9	–0.04	0.19	yes (0.97)	***0.861***
		upd3 (*n* = 3)	gut, –RU	1.0				
			gut, +RU (10)	0.8	–0.12	0.09	yes (0.95)	***0.317***
		aos (*n* = 3)	gut, –RU	1.0				
			gut, +RU (10)	1.3	0.12	0.24	yes (0.84)	***0.667***
		rho (*n* = 3)	gut, –RU	1.0				
			gut, +RU (10)	2.1	0.33	0.40	no (0.041)	***1.000***
		puc (*n* = 3)	gut, –RU	1.0				
			gut, +RU (10)	1.4	0.14	0.23	yes (0.17)	***0.602***

S1 Table. *qPCR* data (Continued).

Figure	Genotype	Gene	Probe	Expression (fold change [a])	$\log_{10}$ (fold change)	SEM ($\log_{10}$)	Normal distribution [b]	*P*-value [c]
**S1B**	*white*	*Hsp70A*	gut, –RU	1.0				
		(*n* = 3)	gut, +RU (10)	0.9	–0.04	0.32	yes (0.83)	***0.918***
		Irc	gut, –RU	1.0				
		(*n* = 3)	gut, +RU (10)	0.8	–0.10	0.04	yes (0.42)	***0.133***

[a] Geometric mean of fold changes. [b] Shapiro-Wilk test. [c] One-sample t-test.

AB, antibiotics treatment; *aos*, *argos*; *Dro*, *Drosocin*; *Hsp70A*, *Heat-shock-protein-70A*; *Irc*, *Immune-regulated catalase*; *Pe*, *Pseudomonas entomophila*; *PGRP-LB*, *Peptidoglycan recognition protein LB*; *pirk*, *poor Imd response upon knock-in*; *puc*, *puckered*; *rho*, *rhomboid*; RU, RU treatment (µg/ml); SEM, standard error of the mean; *Socs36E*, *Suppressor of cytokine signaling at 36E*; tai (h), time after infection (in hours); *upd3*, *unpaired 3*.

Genotypes were:
w/y,w;UAS-Dro/+;tubulin^{GeneSwitch}*-gal4/+* (*Tub*^{GS}*>Dro*),
w/y,w;+/+; tubulin^{GeneSwitch}*-gal4/UAS-CecA1* (*Tub*^{GS}*>CecA1*),
w/y,w;UAS-Dro/+; TiGS2^{GeneSwitch}*-gal4/+ (Ti*^{GS2}*>Dro)*,
w/w;+/+; +/+ (*white*).
