# Supplementary material for: Antimicrobial peptides extend lifespan in Drosophila
Source: PLoS One. 2017 May 17;12(5):e0176689. doi: 10.1371/journal.pone.0176689 (PMC5435158; doi:10.1371/journal.pone.0176689)
Supplement: S2 Table — (PDF) [file pone.0176689.s005.pdf]

S2 Table. Lifespan analysis data.

Figure	Genotype	Treatment	*n* [a]	Mean lifespan (days)	Median lifespan (days)	*P*-value [b]
**1B**	*Tub^GS^>Dro*	–RU	220 (11)	32.5	32	
		+RU (1)	221 (24)	36.4	36	***< 0.001***
Per cent extension				***12.0 %***	***12.5 %***	
**1C**	*Tub^GS^>CecA1*	–RU	191 (10)	32.7	31	
		+RU (1)	222 (10)	35.1	35	***0.020***
Per cent extension				***7.3 %***	***12.9 %***	
**1G**	*Ti^GS2^>Dro*	–RU	174 (1)	26.5	26	
		+RU (10)	169 (7)	28.5	28	***< 0.001***
Per cent extension				***7.5 %***	***7.7 %***	
**2C**	*Tub^GS^>Dro*	–RU, –AB	220 (11)	32.5	32	
		+RU (1), –AB	221 (24)	36.4	36	*see 1B*
		–RU, +AB	217 (12)	36.9	37	***< 0.001*** [c]
		+RU (1), +AB	221 (12)	36.1	37	***0.089*** [d]
Per cent extension				***13.5 %*** [e]<br>***–2.2 %*** [f]	***15.6 %*** [e]<br>***0.0 %*** [f]	
**2D**	*Ti^GS2^>Dro*	–RU, –AB	174 (1)	26.5	26	
		+RU (10), –AB	169 (7)	28.5	28	*see 1G*
		–RU, +AB	218 (20)	30.0	29	***< 0.001*** [c]
		+RU (10), +AB	223 (23)	30.2	30	***0.935*** [d]
Per cent extension				***13.2 %*** [e]<br>***0.7 %*** [f]	***11.5 %*** [e]<br>***3.4 %*** [f]	
**S1A**	*Tub^GS^>Dro*	–RU	111 (4)	30.0	30	
		+RU (1)	112 (5)	33.3	33	***< 0.001***
Per cent extension				***11.0 %***	***10.0 %***	
**S1B**	*Tub^GS^>Dro*	–RU	111 (4)	30.0	30	
		+RU (0.5)	109 (5)	33.2	32	***< 0.001***
Per cent extension				***10.7 %***	***6.7 %***	
**S1C**	*Tub^GS^>Dro*	–RU	105 (13)	32.5	33	
		+RU (10)	113 (13)	31.1	31	***0.192***
Per cent extension				***–4.3 %***	***–6.1 %***	
**S1D**	*Tub^GS^>CecA1*	–RU	221 (8)	33.7	34	
		+RU (1)	217 (10)	34.9	35	***0.019***
Per cent extension				***3.6 %***	***2.9 %***	
**S1E**	*Tub^GS^*	–RU	218 (8)	35.3	35	
		+RU (0.5)	218 (8)	35.0	35	***0.279***
		+RU (1)	219 (10)	35.6	35	***0.989***
Per cent extension				***–0.8 %*** [g]<br>***0.8 %*** [h]	***0.0 %*** [g]<br>***0.0 %*** [h]	
**S1F**	*white*	–RU	221 (3)	27.2	26	
		+RU (0.5)	219 (3)	27.0	27	***0.310***
		+RU (1)	220 (2)	26.7	25	***0.181***
Per cent extension				***–0.7 %*** [g]<br>***–1.8 %*** [h]	***3.8 %*** [g]<br>***–3.8 %*** [h]	
**S1G**	*Ti^GS2^>Dro*	–RU	152 (0)	35.7	36	
		+RU (10)	146 (5)	37.0	37	***0.010***
Per cent extension				***3.6 %***	***2.8 %***	
**S1H**	*Ti^GS2^>Dro*	–RU	433 (2)	28.0	28	
		+RU (10)	433 (7)	29.6	29	***< 0.001***
Per cent extension				***5.7 %***	***3.6 %***	
**S1I**	*Ti^GS2^>Dro*	–RU	130 (10)	27.9	27	
		+RU (10)	133 (11)	30.6	29	***0.003***
Per cent extension				***9.7 %***	***7.4 %***	

S2 Table. Lifespan analysis data (Continued).

Figure	Genotype	Treatment	*n* [a]	Mean lifespan (days)	Median lifespan (days)	*P*-value [b]
**S1J**	*Ti^{GS2}>Dro*	–RU	130 (10)	27.9	27	
		+RU (1)	129 (11)	28.2	27	***0.931***
Per cent extension				***1.1 %***	***0.0 %***	
**S1K**	*Ti^{GS2}*	–RU	173 (13)	28.6	29	
		+RU (10)	176 (16)	27.8	28	***0.493***
Per cent extension				***–2.8 %***	***–3.4 %***	
**S1L**	*white*	–RU	112 (0)	29.2	29	
		+RU (10)	113 (0)	30.5	29	***0.616***
Per cent extension				***4.5 %***	***0.0 %***	
**S2B**	*white*	–AB	108 (0)	37.9	38	
		+AB	109 (2)	40.7	41	***0.010*** [i]
Per cent extension				*7.4 %*	*7.9 %*	

[a] Total number flies (censored flies); [b] Log-rank test; [c] compared to –RU, –AB; [d] compared to –RU, +AB; [e] –RU, +AB vs. –RU, –AB; [f] +RU, +AB vs. –RU, +AB; [g] +RU (0.5) vs. –RU; [h] +RU (1) vs. –RU; [i] compared to –AB.

AB, antibiotics treatment; +RU, RU treatment (concentration in µg/ml).

Genotypes were:
w/y,w;UAS-Dro/+;tubulin^{GeneSwitch}-gal4/+ (*Tub^{GS}>Dro*),
w/y,w;+/+; tubulin^{GeneSwitch}-gal4/UAS-CecA1 (*Tub^{GS}>CecA1*),
w/y,w;UAS-Dro/+;TiGS2^{GeneSwitch}-gal4/+ (Ti^{GS2}>Dro),
w/y,w;+/+;tubulin^{GeneSwitch}-gal4/+ (*Tub^{GS}*),
y,w/y,w;+/+; TiGS2^{GeneSwitch}-gal4/TiGS2^{GeneSwitch}-gal4 (Ti^{GS2}),
w/w;+/+;+/+ (*white*).
