# Supplementary material for: Antimicrobial peptides extend lifespan in Drosophila
Source: PLoS One. 2017 May 17;12(5):e0176689. doi: 10.1371/journal.pone.0176689 (PMC5435158; doi:10.1371/journal.pone.0176689)
Supplement: S3 Table — (PDF) [file pone.0176689.s006.pdf]

S3 Table. CFU analysis data.

Figure	Genotype	Treatment	*n* [a]	CFU (*Pe*) [b]	SEM			Normal distribution [c]	*P*-value [d]
**2A**	*Tub^{GS}>Dro*	4 h pi, +*Pe,* –RU	16 (2)	4.04E+08	5.17E+07			yes (0.43)	
		4 h pi, +*Pe,* +RU (0.5)	19 (2)	2.36E+08	1.90E+07			yes (0.56)	***0.001***
		4 h pi, +*Pe,* +RU (1)	18 (2)	2.54E+08	3.20E+07			yes (0.50)	***0.005***
**2B**	*Ti^{GS2}>Dro*	4 h pi, +*Pe,* –RU	15 (2)	2.74E+08	2.55E+07			yes (0.60)	
		4 h pi, +*Pe,* +RU (10)	16 (2)	2.00E+08	1.64E+07			yes (0.18)	***0.019***
Figure	Genotype	Treatment	*n* [e]	CFU (gut microbiota) [b]	SEM			Normal distribution [c]	*P*-value
**2G**	*Ti^{GS2}>Dro*	–RU (Ace plates)	3	499.7	205.6			yes (0.09)	
		+RU (10) (Ace plates)	3	466.9	163.3			no (0.001)	***1.000*** [f]
		–RU (LB plates)	3	627.0	316.9			yes (0.21)	
		+RU (10) (LB plates)	3	567.8	187.2			yes (0.07)	***0.880*** [g]
Figure		Treatment	*n* [h]	CFU (*Pe*) [i]	SEM	log_{10} (CFU)	SEM (log_{10})	Normal distribution [c]	*P*-value [j]
**S1A**		–RU (LB plates)	3	1.00					
		+RU (1) (LB plates)	3	1.02	0.02	0.01	0.03	yes (0.78)	***0.840***
		+RU (10) (LB plates)	3	0.89	0.03	–0.05	0.04	yes (0.13)	***0.347***
		+RU (100) (LB plates)	3	0.96	0.03	–0.02	0.05	yes (0.71)	***0.729***
Figure		Treatment	*n* [h]	CFU (gut microbiota) [i]	SEM	log_{10} (CFU)	SEM (log_{10})	Normal distribution [c]	*P*-value
**S1C**		–RU (Ace plates)	3	1.00					
		+RU (1) (Ace plates)	3	0.91	0.02	–0.04	0.03	yes (0.76)	***0.301*** [j]
		+RU (10) (Ace plates)	3	1.12	0.02	0.05	0.03	yes (0.61)	***0.211*** [j]
		+RU (100) (Ace plates)	3	0.89	0.03	–0.05	0.04	yes (0.58)	***0.370*** [j]
		–RU (LB plates)	3	1.00					
		+RU (1) (LB plates)	3	1.04	0.03	0.02	0.05	yes (0.97)	***0.748*** [j]
		+RU (10) (LB plates)	3	0.96	0.01	–0.02	0.02	no (0.01)	***1.000*** [k]
		+RU (100) (LB plates)	3	1.10	0.02	0.04	0.03	yes (0.15)	***0.260*** [j]

[a] Total number of guts (number of experiments). [b] Mean CFU per gut. [c] Shapiro-Wilk test. [d] ANOVA (Fisher´s LSD test) for figure 2A, two-sample t-test for figure 2B. [e] Number of experiments (each experiment with 10 guts). [f] Mann-Whitney test. [g] Two-sample t-test. [h] Number of experiments. [i] Mean (geometric) relative CFU. [j] One-sample t-test. [k] Wilcoxon signed-rank test.

AB, antibiotics treatment; CFU, colony forming units; h pi, hours post infection; *Pe*, *Pseudomonas entomophila*; RU, RU treatment (concentration in μg/ml); SEM, standard error of the mean.
