# Supplementary material for: Antimicrobial peptides extend lifespan in Drosophila
Source: PLoS One. 2017 May 17;12(5):e0176689. doi: 10.1371/journal.pone.0176689 (PMC5435158; doi:10.1371/journal.pone.0176689)
Supplement: S4 Table — (PDF) [file pone.0176689.s007.pdf]

S4 Table. Smurf analysis data.

Figure	Genotype (age)	Treatment	*n* [a]	Smurf	No Smurf	% Smurf	*P*-value [b]
3C	*Ti*^{GS2}>*Dro*	–RU	88	0	88	0.0	
	(21 d)	+RU (10)	88	0	88	0.0	n.d.
	Ti^{GS2}>*Dro*	–RU	87	5	82	5.7	
	(28 d)	+RU (10)	88	0	88	0.0	***0.029***
	Ti^{GS2}>*Dro*	–RU	66	10	56	15.2	
	(35 d)	+RU (10)	70	3	67	4.3	***0.041***
	Ti^{GS2}>*Dro*	–RU	30	7	23	23.3	
	(43 d)	+RU (10)	33	4	29	12.1	***0.325***
S3C	*Ti*^{GS2}>*Dro*	–RU	133	0	133	0.0	
	(21 d)	+RU (10)	128	0	128	0.0	n.d.
	Ti^{GS2}>*Dro*	–RU	132	3	129	2.3	
	(28 d)	+RU (10)	128	0	128	0.0	***0.247***
	Ti^{GS2}>*Dro*	–RU	175	35	140	20.0	
	(35 d)	+RU (10)	183	10	173	5.5	***< 0.001***
	Ti^{GS2}>*Dro*	–RU	102	14	88	13.7	
	(43 d)	+RU (10)	116	16	100	13.8	***1.000***

[a] Number of flies. [b] Fisher´s exact test. n.d., not determined.
